# Health Status of Skopelos Goats and Its Impact on Milk Yield Under Intensive and Extensive Farming Systems

**DOI:** 10.3390/ani15091328

**Published:** 2025-05-04

**Authors:** Vera Korelidou, Aphrodite I. Kalogianni, Georgios Arsenos, Athanasios I. Gelasakis

**Affiliations:** 1 Laboratory of Anatomy and Physiology of Farm Animals, Department of Animal Science, School of Animal Biosciences, Agricultural University of Athens (AUA), Iera Odos 75 Str., 11855 Athens, Greece; vkorelidou@aua.gr (V.K.); afrokalo@aua.gr (A.I.K.); 2 Laboratory of Animal Production and Environmental Protection, Faculty of Veterinary Medicine, Aristotle University of Thessaloniki, 54124 Thessaloniki, Greece; arsenosg@vet.auth.gr

**Keywords:** health and welfare indicators, dairy goats, milk, farming system, measures of morbidity, evidence-based farming, production loss, longitudinal study

## Abstract

Health and welfare are fundamental to goat production; however, their impact on milk yield in goats reared under different farming systems remains insufficiently studied. The objective was to examine the frequency of various health issues in dairy goats reared under two different farming systems and to assess their effects on daily milk yield (DMY). A total of 286 Skopelos goats from an intensive (N_A_ = 153) and an extensive (N_B_ = 133) farm, all with the same genetic background, were prospectively monitored for two consecutive lactation periods. Each goat was clinically examined for animal-based health indicators every 50 days, while individual DMY was also recorded. Morbidity frequency measures (cumulative incidence, point, and period prevalence) were calculated for each one of the studied health issues, and mixed linear regression models were developed to assess the effect of each one of the studied health issues on DMY. The results indicated that overgrown hooves were more common in intensively reared goats, while anemia and nasal discharge prevailed in extensively reared goats. Udder asymmetry, udder fibrosis, body abscesses, poor hair coat quality, and swollen lymph nodes were common in both farms, albeit more prevalent in extensively reared goats, except for udder asymmetry. Among the studied health issues, lameness (18.5%, *p* < 0.05), mouth lesions (14.1%, *p* < 0.05), udder asymmetry (6.6%, *p* < 0.001), and udder fibrosis (9.5%, *p* < 0.001), were significantly associated with reductions in DMY. These findings provide valuable insights for optimizing herd health management practices in dairy goats based on evidence and farming system requirements.

## Simple Summary

Animal health and welfare are crucial aspects of livestock production, impacting animal well-being and productivity, as well as farm sustainability. Currently, several health and welfare indicators have been proposed for assessing the health and welfare status of goats. However, their applicability across different farming systems, as well as their role in daily milk yield, have been insufficiently studied. The objective here was to determine the most prevalent health and welfare issues and quantify their impact on the daily milk yield of Skopelos dairy goats that shared the same genetic background but were reared under different farming systems, intensive or extensive. In intensively reared goats, health and welfare issues were related to housing conditions, hygiene standards, and dietary practices. However, in extensively reared goats, grazing activity, parasitism, and lack of systematic health monitoring were the dominant factors of health and welfare issues observed. Among the studied health and welfare issues, lameness, mouth lesions, udder fibrosis, and udder asymmetry were associated with decreased milk production. The results suggest that tailored management practices and preventive measures should be implemented based on evidence and the specific demands of each farming system to optimize animal health, welfare, and productivity.

## 1. Introduction

In Greece, dairy goat farming has a long-standing tradition, especially in less favored, mountainous, rural areas, where land availability for crop production and other agricultural activities is limited. It is also the largest dairy goat population in Europe, and goat milk is primarily used with sheep milk for producing Feta cheese—a protected designation of origin (PDO) product [1,2].

Greek goat farming systems have been classified into four distinct typologies based on shared management practices, resource use, and production strategies. These include (i) large, intensive, semi-intensive, and high-output systems that leverage economies of scale and make limited use of grazing, (ii) traditional extensive and semi-extensive systems characterized by low-input management, limited infrastructure, and all-year-round grazing utilizing natural resources, (iii) medium-sized semi-intensive systems that typically have low replacement rates and invest in land use for forage and feed production, and (iv) low-input extensive and semi-extensive systems primarily focused on producing heavy-weight kid carcasses [3]. The majority of goats in Greece, including Skopelos, are reared under extensive and semi-extensive, low-input farming systems. However, the increasing demand for goat milk over the last two decades, along with the need for enhanced productivity and economic viability, has led to the intensification of farming systems, a trend expected to become dominant in the future [3,4,5].

Regardless of the farming system, health and welfare issues are dominant challenges undermining the sustainability of goat farms [4,6,7,8]. The associations between farming systems and the health and welfare status of dairy goats have been studied [9,10,11], and several protocols, including animal- or herd-level indicators, have been developed [7,9,12,13,14,15].

According to the European Food Safety Authority (EFSA) [16], the assessment of the health and welfare status of farm animals should prioritize animal-based indicators over resource- or farm-based ones. The implementation of validated welfare protocols, including animal-based indicators related to feeding, health, housing, and behavior, enables early and non-invasive detection of potential health issues [17]. Regular assessment of these indicators depicts animals’ response to farming systems and facilitates sustainable farming practices through timely interventions that reduce economic losses and improve overall herd management [18]. The notion is that the latter approach benefits animal health and welfare status and supports the economic viability of farms by maintaining high-quality production while minimizing losses due to disease or poor management. However, except for low body condition scores (BCSs) [19,20,21] and mastitis [22,23,24], there is scarce evidence in the available literature regarding the impact of other health and welfare issues on milk production in dairy goats, particularly when raised under different farming systems [25,26,27,28]. Mastitis, defined as the inflammation of the mammary gland, is among the most studied health issues in various dairy goat breeds (e.g., Murciano-Granadina, Shami × Anglo Nubian, Saanen × Anglo Nubian, Skopelos) [22,29,30]. It has been frequently associated with production losses and degraded milk quality [31].

Hence, the objective of the present study was twofold: (i) to prospectively assess the health and welfare status of Skopelos goats reared under intensive and extensive farming systems using animal-based indicators, and (ii) to estimate the associations between the observed health and welfare issues and milk yield.

## 2. Materials and Methods

### 2.1. Farms and Animals Involved

A total of 286 purebred Skopelos goats were randomly selected from an intensive goat farm (Farm A) (N_A_ = 153) and an extensive goat farm (Farm B) (N_B_ = 133) and were monitored prospectively for two consecutive lactation periods between February 2022 and August 2023. Farm A is in the suburbs of Athens, Greece, 200 m above sea level, with a mild Mediterranean climate and ambient temperatures ranging from −4 to 40 °C. Farm B is located on the island of Skopelos, 50 m above sea level, with a mild Mediterranean climate characterized by dry summers, rainy winters, and ambient temperatures ranging from 5 to 34 °C. All goats were at the same stage of lactation and had the same genetic merit; goats of Farm A were originally purchased from Farm B three years before the study began. The structural and management characteristics of each farm, as reported by the farmers before the initiation of the study, are summarized in Table 1.

### 2.2. Data Recording

**Figure 1:**
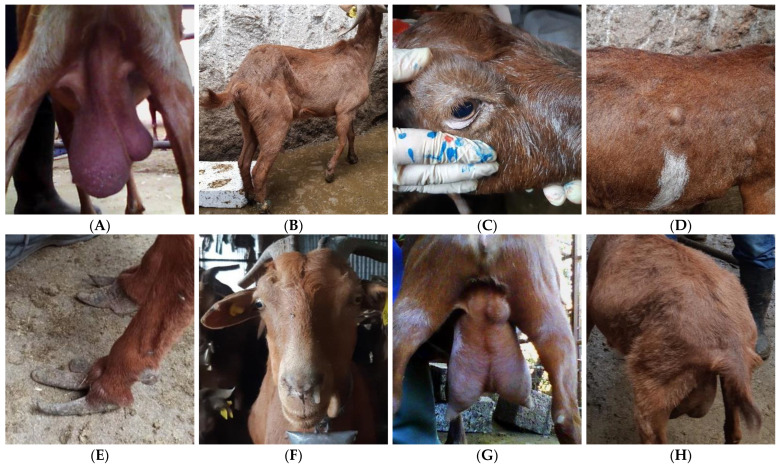
Health and welfare issues identified during the clinical examination of Skopelos goats, namely: severe udder asymmetry (**A**), lameness (**B**), anemia—pale mucus membrane (**C**), multiple body abscesses (**D**), overgrown hooves (**E**), nasal discharge (**F**), udder abscess (**G**), poor hair coat quality (**H**). (Source: Laboratory of Anatomy and Physiology of Farm Animals, Agricultural University of Athens).

Recording sessions (RSs) were initiated at post-weaning and then every 50 days, resulting in four RS per year: RS1 in February (20 days post-weaning), RS2 in April (70 days post-weaning), RS3 in June (120 days post-weaning), and RS4 in August (170 days post-weaning). These sessions included a detailed recording of individual goat health status and milk yield. The evaluation of goat health status was performed by the same veterinarian using a modified version of the AWIN protocol (Animal Welfare Indicators) for dairy goats [32]. Each goat was restrained and subjected to a clinical examination to assess the following: (i) head: signs of anemia, mouth lesions, jaw swellings, nasal and ocular discharges, and swollen parotid and submandibular lymph nodes, (ii) limbs: lameness, arthritis, and overgrown hooves, (iii) udder and teats: cysts, lesions, abscesses, signs of acute clinical intramammary infection, fibrosis, asymmetry, and swollen supra-mammary lymph nodes, and (iv) body: BCS, fecal soiling, poor hair coat quality, vaginitis, meteorism, abscesses, and swollen lymph nodes (prefemoral, prescapular); occurrence of cough was also assessed. Prefemoral, prescapular, parotid, and submandibular lymph nodes were not evaluated during the first RS of both years in both farms, while anemia was not assessed in the first RS of the first year in both farms. Table 2 summarizes the recorded health indicators, while some of the health and welfare issues observed are illustrated in Figure 1. The day after the clinical examination, milk yield was recorded during the morning milking session using a volumetric milk production measuring system, followed by ICAR (International Committee for Animal Recording) guidelines for calculating the 24 h milk yield from a single milking [33]. The experimental protocol was approved under authorization number 33/8.06.21, granted by the Ethics Committee of the Agricultural University of Athens.

### 2.3. Statistical Analysis

Morbidity frequency measures, including point prevalence (the proportion of goats with the observed health issue at a specific point in time), period prevalence (the proportion of goats with the observed health issue at any time during the specified period), and cumulative incidence (CI) (the proportion of goats at risk at the start of the observation period that developed the observed health issue during the specified period), were calculated for the studied health and welfare issues. For the estimation of CI, only goats with a full dataset of recordings were considered per recording year. Among the 153 goats enrolled in this study from Farm A, complete datasets from all four RS were available for 100 goats in the first year and 103 goats in the second year. Similarly, in Farm B, CI values were estimated based on data collected from 115 goats in the first year and 81 goats in the second year. Descriptive statistics (mean ± standard deviation) of daily milk yield (DMY) and BCS were also calculated throughout the study. Finally, 24 mixed linear regression models, one for each of the studied health issues, were built to assess their effects on DMY as described below (Equation (1)). Data was analyzed using SPSS v.26 software (IBM Corp., Armonk, NY, USA), with statistical significance set at the a = 0.05 level, while a statistical significance level of 0.001 was used when necessary:

DMΥ_gj_ = μ + F_j_ + Y_gj_ + A_gj_ + S_j_ + Χ_gj_ + β_1_ × ΒCS + γ_j_ + e_gj_ (1)

where DMY_gj_ = daily milk yield (kg) for the gth sampling occasion of the jth goat, μ = intercept, F_j_ = fixed effect of the farming system (2 levels; 1 = intensive, 2 = extensive), Y_gj_ = fixed effect of the year of sampling (2 levels; 1 = 1st, 2 = 2nd year), A_gj_ = fixed effect of age (4 levels; 1 = 2, 2 = 3, 3 = 4, and 4 ≥ 5 years old), S_j_ = fixed effect of the stage of lactation (4 levels; 1 = 20, 2 = 70, 3 = 120, and 4 = 170 days post-weaning), X_gj_ = fixed effect of the occurrence of the recorded health issues (2 levels; 0 = absence, 1 = presence), β_1_ = fixed effect of the regression coefficient of BCS (1 to 5 with 0.25 increments), γ_j_ = repeated variation of the jth goat, and e_gj_ = residual error.

## 3. Results

### 3.1. Morbidity Frequency Measures

Table 3 and Table 4 illustrate the point and period prevalence values for each one of the studied health and welfare issues in Farms A and B during the first and second year of recordings, respectively. The number of new cases and the CI values for studied health and welfare indicators in Farms A and B are presented in Table 5 and Table 6, respectively.

Hoof overgrowth was the most common foot-related issue observed, with point prevalence values ranging from 1.0 to 31.4% in Farm A and from 0.0 to 3.0% in Farm B. Arthritis and lameness were less frequent (point and period prevalence < 4.0%) and primarily observed in the intensively reared goats. Most lameness and overgrown hoof cases were observed during the first and second RSs. Moreover, the total number of goats with overgrown hooves in Farm A increased from 29.2 to 42.1% in the second year, while the period prevalence values for arthritis and lameness remained at the same levels.

Anemia was observed throughout lactation in both farms; however, it was more prevalent in Farm B compared to Farm A. Similarly, nasal discharge was more common in Farm B than in Farm A, with the highest point prevalence values observed in RS4. In addition, in Farm B, distinct seasonal patterns were observed for anemia and nasal discharge; specifically, new cases of anemia declined as summer approached, while the occurrence of nasal discharge increased. Total cases of anemia and nasal discharge increased from 48.9 to 71.9% and from 12.8 to 43.9%, respectively, in Farm B in the second year of the study. Conversely, in Farm A, the total cases of anemia remained stable, while nasal discharge was particularly evident in the RS4 of the second year.

An increase in the number of goats with mouth lesions was observed in Farm A, particularly in the second RS of the first year, ultimately affecting 22.6% of the goats. Jaw swellings were consistently observed in Farm A but at low-point prevalence values (<6.0%). Finally, ocular discharge was occasionally found in both Farms A and B.

The clinical examination of the udder indicated that udder asymmetry, udder fibrosis, and swollen supra-mammary lymph nodes were among the most frequent udder health-related issues observed in both farms. In particular, udder fibrosis and swollen supra-mammary lymph nodes were observed at higher CI and point and period prevalence values in Farm B compared to Farm A, while morbidity frequency measures did not differ for udder asymmetry between the two farms. Furthermore, in the second year of recordings, a remarkable increase from 14.2 to 45.5% and from 24.1 to 51.8% was observed in Farm A and Farm B, respectively, for swollen supra-mammary lymph nodes. On the contrary, the total number of goats displaying udder asymmetry decreased from 74.5 and 75.2% to 44.1 and 45.6% in Farm A and Farm B, respectively.

Teat and udder cysts, as well as udder abscesses, were primarily found in Farm B, albeit at low point and period prevalence values (<10.0%), with no remarkable difference between the two years, while signs of acute clinical intramammary infection, teat fibrosis, and udder skin lesions were less frequently observed in both farms (point and period prevalence values ≤ 3.0%).

Poor hair quality along with swollen body lymph nodes was among the most common health-related issues identified throughout the study, with point prevalence values ranging from 3.9 to 31.0% and 15.4 to 28.6%, respectively, in Farm A and from 7.1 to 47.0% and 29.5 to 54.5%, respectively, in Farm B. In both farms, poor hair coat quality was mostly observed at the beginning of the lactation period (RS1). The period prevalence of poor hair coat quality decreased in both farms (from 48.1 to 30.3% in Farm A and from 60.9 to 51.8% in Farm B), while the period prevalence of swollen body lymph nodes increased in Farm B from 45.9 to 64.9% in the second year of the study.

Body abscesses were also common in both farms, with point prevalence values ranging from 8.0 to 35.9% in Farm A and from 14.9 to 27.3% in Farm B. Fecal soiling, vaginitis, meteorism, and cough were occasionally found in both farms, with the last three observed at point and period prevalence values < 5.0%, while no case of dyspnoea was recorded at the time of examination.

### 3.2. Milk Yield and Body Condition Score Variation Throughout Lactation Period

**Figure 2:**
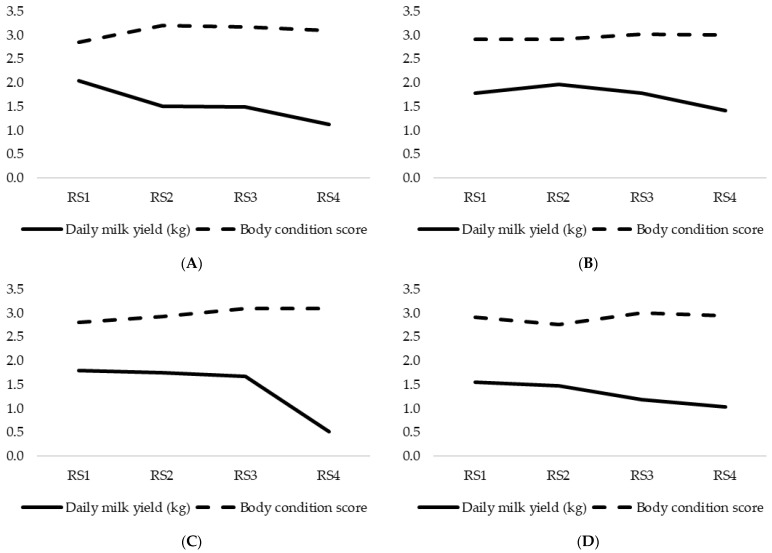
Average daily milk yields (kg) and body condition scores (BCSs, 1–5) in the intensive farm, in the first ((**A**), N_A_ = 106) and second ((**B**), N_B_ = 146) year of recordings, and in the extensive farm, in the first ((**C**), N_C_ = 133) and second year ((**D**), N_D_ = 115) of recordings. The figure illustrates a declining trend in milk yield over lactation in both farms, with higher milk yield in intensive farm and an inverse relationship between milk yield and BCS. (RS1: February (20 days post-weaning), RS2: April (70 days post-weaning), RS3: June (120 days post-weaning), RS4: August (170 days post-weaning)).

In the first year, the average DMY of goats in Farm A gradually decreased from 2.04 ± 0.770 kg at the start of the milking period to 1.13 ± 0.475 kg in RS4 (Figure 2). In the second year, DMY initiated at 1.79 ± 0.688 kg in the first RS, peaked at 1.96 ± 0.863 kg in the second RS, and then gradually decreased to 1.41 ± 0.750 kg shortly before the end of the lactation period. The average BCSs of goats in Farm A ranged from 2.85 ± 0.191 to 3.22 ± 0.327 in the first year of RS and from 2.91 ± 0.432 to 3.02 ± 0.410 in the second year of RS (Figure 2). In Farm B, the average DMY varied between 1.80 ± 0.532 and 0.51 ± 0.244 kg in the first year and between 1.55 ± 0.483 and 1.04 ± 0.434 kg in the second year. The mean BCS steadily increased from 2.80 ± 0.154 to 3.10 ± 0.293 over the first year, while in the second year, the BCS decreased from 2.91 ± 0.270 to 2.76 ± 0.319 in the second RS before showing a slight increase in the third RS.

### 3.3. Mixed Linear Regression Models

A total of 24 mixed linear regression models were used to assess the impact of various health issues on DMY (Table 7). Significant reductions in milk yield were observed in goats with the following conditions: lameness (18.5%, *p* < 0.05), mouth lesions (14.1%, *p* < 0.05), udder fibrosis (9.5%, *p* < 0.001), and udder asymmetry (6.6%, *p* < 0.001). Additionally, the presence of overgrown hooves showed a tendency towards reduced DMY (*p* = 0.059). Although the highest decrease in DMY was associated with cough, this effect was not statistically significant (*p* = 0.127). In all cases, there was a positive association between age and milk yield (*p* < 0.001), indicating that older goats produced more milk. Conversely, both the stage of lactation and BCS showed a negative correlation with milk yield (*p* < 0.001). Lastly, intensively reared goats produced approximately 0.5 kg more milk compared to extensively reared ones (*p* < 0.001).

## 4. Discussion

Herein, we prospectively investigated the most common health and welfare issues in goats reared under different farming systems, assessed their frequency, and examined their impact on milk production over a two-year period. To our knowledge, this is the first study to compare the health and welfare status of dairy goats sharing the same genetic background but reared under intensive and extensive farming systems. Additionally, this study adds to the existing literature by analyzing the relationship between a total of 24 health and welfare indicators and milk yield while also providing insights and updating information regarding the health and productivity of Skopelos goats, the only officially recognized purebred Greek goat [8].

In our study, health and welfare issues related to feet were more common in intensively reared goats. This is consistent with other studies, where lameness and hoof overgrowth have been identified as prevalent health issues in goats reared under intensive farming systems, with reported prevalence values ranging from 1.2 to 19.2% and from 35.5 to 79.8%, respectively [7,14,15]. Housing conditions and management practices, such as irregular foot trimming, high stocking density, and poor hygiene inside the pen due to excessive accumulation of manure and urine, were likely associated with the higher occurrence of foot-related issues in intensively reared goats, particularly during winter and spring. Contrarily, in extensively reared goats, grazing on rough surfaces facilitates natural hoof wear and promotes the mechanical cleaning of the hoof. Indeed, walking on pasture was associated with improved gait scores [34] and a low occurrence of foot-related issues [35,36].

Lameness has been widely studied in dairy cattle [37]; however, the available literature on the association between lameness and milk production in goats remains limited. Lameness is considered the second costliest health issue in cattle after mastitis [38], leading to reduced milk yield [39], decreased reproductive capacity [40], and high culling rates [41]. The negative effect of lameness on milk yield was confirmed with an 18.0% decrease in DMY of lame goats in the studied population. The financial impact of these losses is estimated at EUR 0.22 per goat, based on the price of goat milk in Greece (0.92 EUR/kg) [42]. Similarly, Jaques et al. [43] observed that severe lameness in seasonal and extended lactating goats led to a reduction of 7.1% and 8.7% in milk yield, with economic losses estimated at 0.28 EUR/goat (0.52 NZD) and 0.21 EUR/goat (0.39 NZD), respectively. In dairy sheep, lameness was associated with a 16.0% (0.21 kg) reduction in milk yield, equivalent to a 50 kg decrease per lactation, resulting in financial losses of EUR 45 per case and treatment costs of EUR 15–20 per case [44].

Among udder health issues in dairy ruminants, mastitis due to intramammary infections (IMI) is the most widely studied due to its significant impact on animal welfare and milk production [45]. In goats, the majority of IMI are subclinical [46]; however, over time, these infections can become clinical, resulting in structural damage within the mammary gland, such as the development of fibrotic tissue, asymmetry, abscesses, and secondary milk cysts [47]. Studies investigating milk yield losses associated with subclinical IMI in dairy goats have led to equivocal results. While some authors have noticed a decrease in milk production [29], others have found no significant difference between infected and uninfected udders [48,49] or even higher milk yield in affected udders [50]. These variations could be attributed to differences in study designs (udder half vs. goat level), as well as to the causative pathogens and their prevalence [23,29]. Intramammary infections in Skopelos and indigenous Greek goat breeds reared under low-input farming systems have been primarily attributed to coagulase-negative Staphylococci and associated with a deterioration in both milk quality and quantity [22,31]. Specifically, goats with IMI produced 5.7% (0.05 kg/day) less milk, with losses increasing to 15.0% (0.12 kg/day) when infected with gram-negative bacteria [22]. In addition, clinical examination of these populations indicated that udder asymmetry was the most repeatable udder health-related issue, while a negative correlation between milk yield and udder asymmetry was confirmed when including Damascus goats [51]. In our study, the estimated milk yield and monetary losses accounted for by udder asymmetry and udder fibrosis were approximately 0.1 kg and EUR 0.08 in both cases, respectively, without accounting for the cost of treatment. In Alpine and Saanen goats, severe asymmetry was associated with a 14.1% (100 kg) decrease in milk yield, shortened lactations (<250 days), higher SCC, and higher culling rates, while mild asymmetry led to a 4.9% (37.8 kg) decrease in milk yield [52].

Udder asymmetry is among the most prevalent udder health-related issues in dairy goats [7], with prevalence ranging from 3.3 to 34.4% [14,15] in intensively reared goats and from 4.8 to 38.2% in extensively or semi-extensively reared goats [35,51]. A CI of 43.0% has been previously reported in goats reared under low-input farming systems [8], which falls within the range observed in our study.

In our study, udder asymmetry was possibly linked to chronic, untreated IMI, resulting in fibrosis and udder atrophy. Both udder fibrosis and udder asymmetry were prevalent throughout the lactation period and were associated with improper milking practices, high stocking density, and poor hygiene in the intensive farm, as well as hand-milking practices and the lack of an udder health management program in the extensive farm (e.g., post-milking dipping, dry antibiotic therapy). Notably, there was no distinct pattern related to the emergence of new cases. However, the prevalence of udder fibrosis increased during the second year in the extensive farm, likely due to the chronic nature of the disease and the absence of proper treatment during the dry period. Indeed, in low-input farming systems, chronic IMI has been associated with the absence of control programs, the low culling rate of infected goats, and the failure to treat IMI during the dry period [22].

Regarding other udder health-related issues, swollen supra-mammary lymph nodes were common in both farms but were slightly more prevalent in extensively reared goats. Similarly, udder and teat cysts, as well as udder abscesses, though having a low prevalence, were more common in extensively reared goats, likely due to hand-milking practices. Skopelos goats reared under low-input farming systems have been found to be susceptible to udder abscesses, yet without any impact on milk yield [51].

An outbreak of lesions (papules and scabs) on the mouth and muzzle was observed during the second recording session of the first year in the intensive farm, resulting in a 14.5% reduction in DMY of the affected goats. This decrease equates to a loss of 0.2 kg and EUR 0.18 per goat. Although several etiological agents could have caused mouth lesions and laboratory confirmation was not available, the clinical presentation and epizootiology of lesions were indicative of contagious ecthyma. Lesions typically resolve within 4 to 6 weeks [53], as observed in our study, and goats develop long-lasting immunity [54]. Infected animals may exhibit decreased milk yield (as confirmed herein) and weight loss due to impaired feeding resulting from painful oral lesions [55]. The economic burden of the disease, including production losses and treatment costs, has been estimated at GBP 4.62 per affected goat [56]. Among the other health-related issues examined in this study, none were found to affect milk yield.

Anemia, nasal discharge, and poor hair coat quality were observed on both farms but were more prevalent in the extensively reared goats. In particular, anemia was one of the most common health issues of extensively reared goats, observed throughout the lactation period. This finding aligns with the results of Silva Salas et al. [57] and Kim et al. [58], who noted that anemia was more prevalent in semi-intensively reared goats and extensively reared cattle, respectively. This difference was likely due to grazing activity and greater exposure to endoparasites (e.g., *Haemonchus contortus*); exposure to other blood-sucking parasites (e.g., lice, fleas) was not confirmed during the clinical examination of the animals. Other factors that could have contributed to the occurrence of anemia include nutritional deficiencies (copper, iron, cobalt), chronic diseases, or other hemoprotozoan diseases (e.g., Babesiosis, Anaplasmosis, Theileriosis) [59,60].

In our study, the number of new anemia cases declined as summer approached, while CI was higher in the second year in extensively reared goats. Similarly, poor hair coat quality was more prevalent during winter across both years and farms, whereas CI decreased in the second year. During winter, nutritional demands rise due to the onset of lactation and the need for thermoregulation [61], as observed in our case. In extensive farms, the nutritional deficiencies resulting from poor grazing material during winter, coupled with the lack of deworming, may have contributed to the observed patterns of anemia and poor hair coat quality. In contrast, in intensive farms, the scarcity of high-quality hay during winter, along with increased feeding competition due to high stocking density, likely led to nutritional deficiencies and a higher prevalence of poor hair coat quality. In intensively reared goats, poor hair coat quality has been reported, with prevalence rates ranging between 8.3 and 36.4% [15,62], primarily attributed to mineral deficiencies (calcium, magnesium, iron, and manganese) rather than gastrointestinal parasitic infections [63]. Moreover, high stocking density, mixing horned and hornless animals, and limited access to feedstuff may predispose housed animals to poor hair coat quality [15]. In extensively reared goats, the prevalence of poor hair coat quality has been observed to reach up to 62.8%. This condition has been associated with seasonal variations in grazing material, prolonged consumption of high-fiber, low-energy content forage, chronic hunger, and increased exposure to parasites [64]. Poor hair coat quality has been identified as an indicator of an animal’s nutritional and health status; specifically, rough and scurfy hair has been associated with a high prevalence of chronic respiratory diseases and low BCSs in goats [18].

A seasonal pattern was also evident for nasal discharge in our study, with the highest prevalence observed in summer and increased period prevalence and CI values in the second year. Possible underlying causes include infectious agents (bacterial, viral, parasitic) and non-infectious agents (powdery feeds, ammonia) [65]. Similar studies have reported a nasal discharge prevalence below 10% [14,36,51] and a CI of 19.9% in Skopelos, Eghoria, and Damascus goats reared under low-input farming systems [8], with significant repeatability in the last two breeds [51].

Body abscesses and swollen lymph nodes were prevalent throughout lactation in both intensively and extensively reared goats. In our study, abscesses were usually located in the lymph nodes, likely due to infections by *Corynebacterium pseudotuberculosis*, a gram-positive bacterium that causes caseous lymphadenitis [66]. The transmission of bacteria occurs horizontally, and their introduction into a farm is facilitated by the purchase of infected animals and the sharing of contaminated equipment [8]. In our study, the common origin of the goats explains the high prevalence in both farms. Additionally, factors such as the use of infected needles and a contaminated environment (as seen in the intensive farm), as well as fighting behavior (as seen in the extensive farm), can predispose animals to infection [8]. Abscesses and swollen lymph nodes have been observed in both intensive (0.4–32.2%) [7,12] and extensive goat farming systems (3.8–19.8%) [36,64]. Notably, Battini et al. [67] found that abscesses were present in 90.0% of the studied farms, emphasizing their widespread occurrence, while Leitte et al. [68] reported a prevalence of 70.0% in meat goats. Body abscesses have been linked to lower BCS and decreased feeding time [69]; however, no association has been found with milk production [51]. In sheep, caseous lymphadenitis has been associated with negative impacts on wool, carcass, skin, meat, milk production, and reproductive performance [70].

Lastly, jaw swellings were observed less frequently but consistently reported in intensive farms. This was possibly due to poor feedstuff quality, causing gum injuries and infections. Fecal soiling, vaginitis, meteorism, ocular discharge, and cough were occasionally found in both farms. Among these, only ocular discharge and fecal soiling are included in health and welfare assessment protocols. Muri et al. [14] reported a high prevalence of ocular discharge in goats (35.6%) due to conjunctivitis and environmental factors (e.g., dust or foreign bodies). In contrast, other studies reported either a total absence [35] or a low prevalence [15] of ocular discharge and fecal soiling.

Unlike other studies that involved multiple assessors [67,71], the assessment of health and welfare status in this study was conducted by the same experienced veterinarian each time to minimize any problems related to interobserver repeatability in scoring. Clinical assessments were performed at the animal level, involving both inspection and palpation to detect any health issues, which can be time-consuming. In many studies, assessments were either conducted at the group level or focused solely on health issues that were easily detectable through observation. However, assessments conducted from a distance may result in the underdiagnosis of health issues, as certain body parts may be less visible, and some health issues may require palpation to be accurately identified. Future studies should consider incorporating laboratory techniques to identify the causative agents of the observed health and welfare issues and analyze relevant data on a disease-specific basis. In addition, the inclusion of animals exhibiting health issues associated with the lower respiratory tract would provide a more comprehensive assessment of respiratory health and its potential impact on productivity.

Although our study involved a limited number of farms, all the goats belonged to the same breed, were at the same stage of lactation, and shared the same genetic background. Additionally, goats were prospectively monitored over two consecutive years at different points during the lactation period. This enabled the analysis of how various health and welfare issues evolve throughout lactation and the identification of periods of increased risk. Previous research on Skopelos, Eghoria, and Damascus goats reared under low-input farming systems has indicated that environmental conditions, feeding and water supply limitations, inappropriate infrastructures, as well as a lack of farmers’ expertise and training in herd management and preventive medicine practices are among the key challenges of these systems [8,51]. These challenges are compatible with those observed in our study, suggesting that certain health and welfare issues (e.g., chronic IMI, nasal discharge, abscesses) in extensive farming systems are particularly diachronic. In any case, including a greater number of farms and diverse breeds in future studies could provide a more comprehensive understanding of the dynamics of health and welfare issues and enhance the validity of our findings.

The findings from this study can be utilized to optimize management practices tailored to a specific farming system. According to our results, in intensive farming systems, management strategies should emphasize (i) adjusting diets based on goats’ nutritional demands and providing high-quality feedstuff, particularly during winter, (ii) maintaining proper hygiene within the pen through regular replacement of bedding material, litter removal, and disinfection, (iii) ensuring good housing conditions, such as a space allowance of 2.0 m^2^ per goat [72] and sufficient airflow, (iv) performing regular foot trimming as required, and (v) applying proper milking practices, maintaining high hygiene standards in the milking parlor, ensuring the good functioning of milking machines, and performing regular assessments of udder health status. In extensive farming systems, management practices should focus on (i) meeting goats’ nutritional demands to prevent deficiencies, (ii) applying good milking practices, and (iii) adopting health management practices, such as regular assessment of udder health status, treatment during the dry period, and regular deworming.

Our findings underpin the demand for evidence-based and efficient herd health management tailored to the peculiarities of the existing farming systems. The systematic monitoring and recording of health and welfare traits by a vet may be subjective, labor-intensive, and impractical under real-world conditions. To address this challenge, precision livestock farming (PLF) technologies have the potential to support farmers in decision-making driven by animal-based indicators. These technologies enable the automated, in situ collection of real-time, continuous, and objective data from animals and facilitate further analyses [73], thereby saving time, allowing early intervention, and enhancing the capacity for individualized animal care [74]. Currently, image and sound-based technologies (e.g., 2D, 3D, and thermal cameras, microphones) combined with deep learning, along with Radio-Frequency Identification (RFID) and wireless communication technologies, as well as sensors either attached to animal bodies or installed within barns (e.g., collars with gps and accelerometers, reticulum boluses, thermometers) [75] have been used for livestock monitoring and management both in intensive and pasture-based farming systems [76]. These technologies have been particularly used for animal tracking and behavior analysis [77,78], monitoring nutritional status [79], physiological animal parameters [80], and physical environment [81], as well as identifying health and welfare-related issues (e.g., lameness, cough, mastitis) [82,83].

## 5. Conclusions

This study examined various health and welfare issues in Skopelos goats reared under intensive and extensive farming systems throughout the lactation period and assessed their impact on milk production. Most health issues in intensively reared goats were primarily linked to housing conditions, hygiene standards, and dietary practices, whereas extensively reared goats faced health issues related to grazing activity, parasitism, and lack of systematic health monitoring. Many of these health issues have been associated with the deterioration of health and welfare. Udder fibrosis, udder asymmetry, lameness, and mouth lesions were observed to decrease milk production. Regular assessment of health and welfare status is crucial for implementing evidence-based management practices that enhance animal welfare and mitigate production losses. Regardless of the farming system, adopting good husbandry practices is crucial for maintaining high health and welfare standards. Including a greater number of farms and breeds, as well as utilizing laboratory diagnostic techniques, could provide further insights into the underlying etiological factors related to the most prevalent health and welfare issues observed herein.

## Figures and Tables

**Table 1:** Structural and management characteristics of farms involved in the study as reported by the farmers.

	Farm A (Intensive Farm)	Farm B (Extensive Farm)
Production		
Milk production/doe/lactation (210 days), kg	450	350
Total annual milk production, tn	75	85
Average milk fat content, %	4.0	4.3
Average milk protein content, %	3.7	3.9
Average somatic cell count (cells/mL)	5 × 10^5^–1 × 10^6^	5 × 10^5^–1 × 10^6^
Management		
Duration of milking period, d	200	150
Duration of suckling period, d	60	90
Type of milking	Machine milking (Milkplan, Herringbone milking parlor 2 × 24 with silicone rubber cups)	Hand-milking
Milking machine parameters	Vacuum level: 40 kPa Pulsation rate: 90 pulses per minute Pulsation ratio: 50% Automatic removal of clusters	Not applicable
Cleaning routine of milking machine after every milking	Rinse with water → alkaline detergent (or acid detergent (twice a week) → water → drying	Not applicable
Number of milkings per day	2 (07:00 and 19:00)	2 (06:00 and 18:00)
Pre-milking preparation	No	No
Milk stripping	Yes	No
Post-dipping of teats	Yes (iodine-based)	No
Reproduction		
Fecundity, *n*	1.6	1.5
Services per conception	≤1.5	≤1.5
Culling rate due to infertility, %	<5	<5
Average litter size at weaning	1.4	1.3
Artificial suckling	Yes	No
Infrastructures and housing		
Type of building	Shed with openings	Shed with openings
Type of floor	Earthen	Earthen
Type of bedding	Straw	No bedding
Ventilation adequacy	Good	Medium
Lighting adequacy	Good	Medium
Type of waterers	Automatic	Barrels
Stocking density, m^2^/goat	1.2	-
Feeding and nutrition		
Feedings per day	2	2
Grazing	No	Yes
Duration of grazing, h	Zero-grazing	8–12
Type of grazing land	Not applicable	Natural grasslands, shrublands/woodlands, cultivated pasturelands
Concentrates in milking goats (16% crude protein, kg/day) *	1.0–1.2	0.5–1.0
Concentrates in dry goats (14% crude protein, kg/day)	0.5	0.5
Roughages (hay) in milking goats (18% crude protein, kg/day) *	0.8–1.9	0.0–0.5
Roughages (hay) in dry goats (17% crude protein, kg/day)	0.4	0.0–0.3
Incidence of health and welfare issues		
Clinical acidosis, %	≤2	≤2
Pregnancy toxemia, %	≤2	≤2
Metritis, %	≤3	≤3
Clinical mastitis, %	≤5	≤5
Subclinical mastitis, %	10–20	10–20
Retained placenta, %	≤2	≤2
Abortion, %	≤3	≤3
Lameness, %	≤5	≤5

* Based on their nutritional demands and stage of lactation.

**Table 2:** Health indicators description and levels of assessment.

	Indicator	Description	Levels
Head	Anemia	Pale mucus membrane	3
	Mouth lesions	Papules and scabs around the mouth and lips	2
	Jaw swelling	Swelling and painful site during palpation at the premolar and molar teeth in the lower or upper jaw	2
	Ocular discharge	Discharges from the eye	2
	Nasal discharge	Discharges from the nasal cavity	2
Limbs	Lameness	Impaired gait	5 *
	Arthritis	Swollen or inflamed limb joints	2
	Overgrown hooves	Excess horn tissue on claws	3
Udder and teats	Cysts	Cysts on udder or teats	2
	Skin lesions	Disrupted integrity of the skin	2
	Abscesses	Swollen, pus-filled cavities	2
	Signs of acute clinical intramammary infections	Painful, hot, hard, and swollen udder	2
	Fibrosis	Hard and fibrotic udder parenchyma at palpation	2
	Asymmetry	Uneven udder halves	3
	Swollen supra-mammary lymph nodes	Enlarged lymph nodes	5 **
Other	Fecal soiling	Manure accumulation below the tail	2
	Poor hair coat quality	Matted, rough, scurfy, uneven, shaggy hair coat	2
	Vaginitis	Inflammation of vagina	2
	Meteorism	Bloated rumen	2
	Body abscesses	Abscess at any part of the body, except for the udder	2
	Swollen body lymph nodes	Enlarged parotid, prescapular, prefemoral, or/and submandibular lymph nodes	5 **
	Body condition score	Nutritional status assessed by the palpation of the lumbar area and the estimation of the fat coverage	5 ***
	Cough	Cough	2
	Abnormal respiration	Dyspnoea	

2 levels: 0 = absence, 1 = presence; 3 levels: 0 = absence, 1 = mild, 2 = severe (values > 0 were considered as presence of the health issue). * 0 = normal, 1 = mild lameness when walking 2 = lameness when walking and occasionally lifting foot when standing, 3 = severe lameness when walking and lifting foot when standing, 4 = carrying foot all time (values > 0 were considered as presence of lameness). ** based on size: 1 = pea, 2 = almond, 3 = nutmeg, 4 = nut, 5 = mandarin or bigger (values > 2 were considered as presence of swollen lymph nodes). *** 5-point scale with 0.25 increments (<2.5: emaciated, 2.5–3.5: normal, >3.5: overweight).

**Table 3:** Point and period prevalence values of the studied health issues in the first year of recordings in Farm A (intensive) and Farm B (extensive).

	Year 1									
	Point Prevalence (%)								Period Prevalence (%)	
	RS1		RS2		RS3		RS4			
	Farm A n = 103	Farm B n = 132	Farm A n = 104	Farm B n = 132	Farm A n = 106	Farm B n = 127	Farm A n = 103	Farm B n = 121	Farm A n = 106	Farm B n = 133
Lameness	0.0	0.0	3.8	0.8	0.0	0.8	1.0	0.8	3.8	1.5
Overgrown hooves	3.0	0.0	21.2	2.3	14.2	1.6	1.0	0.0	29.2	3.8
Anemia	NA	NA	11.5	5.3	1.9	37.0	4.9	35.5	15.1	48.9
Mouth scabs, papules	0.0	0.0	22.1	0.0	0.9	0.0	0.0	0.0	22.6	0.0
Jaw swelling	1.0	0.0	2.9	0.0	5.7	0.8	4.9	1.7	9.4	2.3
Nasal discharge	0.0	0.0	0.0	0.0	4.7	0.8	0.0	13.2	4.7	12.8
Teat cysts	0.0	9.1	0.0	0.8	2.8	3.9	0.0	1.7	2.8	12.0
Udder abscesses	2.0	6.1	1.9	4.5	0.9	1.6	1.0	1.7	4.7	10.5
Udder cysts	0.0	0.8	1.9	3.0	0.0	4.7	0.0	0.0	1.9	8.3
Udder fibrosis	11.0	2.3	19.2	22.7	20.8	35.4	20.4	37.2	44.3	56.4
Udder asymmetry	16.0	10.6	45.2	43.2	51.9	36.2	19.4	47.9	74.5	75.2
Swollen supra-mammary lymph nodes	8.0	13.6	1.9	11.4	7.5	11.0	1.0	10.7	14.2	24.1
Fecal soiling	1.0	0.8	1.9	3.0	0.0	3.9	1.0	2.5	3.8	8.3
Poor hair coat quality	31.0	47.0	8.7	34.8	17.0	31.5	24.3	16.5	48.1	60.9
Body abscesses	8.0	23.5	35.6	22.7	35.8	22.0	33.0	14.9	50.0	45.1
Swollen body lymph nodes (at least one)	NA	NA	15.4	29.5	23.6	33.1	18.4	33.9	36.8	45.9

RS1: February (20 days post-weaning), RS2: April (70 days post-weaning), RS3: June (120 days post-weaning), RS4: August (170 days post-weaning); Farm A: intensive farm, Farm B: extensive farm; NA: not applicable (no recording was performed); period and maximum point prevalence of arthritis, ocular discharge, cough, abnormal respiration, teat fibrosis, signs of acute clinical intramammary infection, udder skin lesions, vaginitis, and meteorism were below 3.0%.

**Table 4:** Point and period prevalence values of the studied health issues in the second year of recordings in Farm A (intensive) and Farm B (extensive).

	Year 2									
	Point Prevalence (%)								Period Prevalence (%)	
	RS1		RS2		RS3		RS4			
	Farm A n = 118	Farm B n = 97	Farm A n = 126	Farm B n = 108	Farm A n = 128	Farm B n = 99	Farm A n = 125	Farm B n = 98	Farm A n = 145	Farm B n = 114
Lameness	2.5	0.0	2.4	0.0	0.0	0.0	0.0	0.0	2.1	0.0
Overgrown hooves	31.4	0.0	20.6	1.9	14.1	3.0	16.8	1.0	42.1	4.4
Anemia	7.6	44.3	6.3	50.0	8.6	46.5	1.6	33.7	16.6	71.9
Jaw swelling	2.5	0.0	1.6	0.9	3.1	0.0	3.2	0.0	5.5	0.9
Nasal discharge	0.0	26.8	0.8	9.3	5.5	5.1	24.8	29.6	25.5	43.9
Teat cysts	0.0	6.2	0.0	4.6	0.0	2.0	0.0	0.0	0.0	7.9
Udder abscesses	2.5	9.3	2.4	0.0	0.8	3.0	0.8	0.0	2.8	9.6
Udder fibrosis	31.4	13.4	22.2	43.5	16.4	39.4	24.0	35.7	48.3	68.4
Udder asymmetry	25.4	11.3	26.2	26.9	24.2	29.3	29.6	20.4	44.1	45.6
Swollen supra-mammary lymph nodes	33.9	27.8	27.0	38.0	22.7	24.2	9.6	17.3	45.5	51.8
Fecal soiling	1.7	0.0	0.8	0.0	0.0	0.0	5.6	1.0	6.9	0.9
Poor hair coat quality	18.6	42.3	17.5	31.5	3.9	19.2	10.4	7.1	30.3	51.8
Body abscesses	28.0	16.5	23.0	19.4	32.0	27.3	28.8	25.5	44.1	38.6
Swollen body lymph nodes (at least one)	NA	NA	28.6	47.2	24.2	54.5	20.0	43.9	40.0	64.9

RS1: February (20 days post-weaning), RS2: April (70 days post-weaning), RS3: June (120 days post-weaning), RS4: August (170 days post-weaning); Farm A: intensive farm, Farm B: extensive farm; NA: not applicable (no recording was performed); period and maximum point prevalence of arthritis, mouth scabs and papules, ocular discharge, cough, abnormal respiration, teat fibrosis, signs of acute clinical intramammary infection, udder skin lesions, udder cysts, vaginitis, and meteorism were below 3.0%.

**Table 5:** New cases and cumulative incidence (total number of new cases/goat population at risk at the beginning) of the studied health and welfare issues per recording session during the first and second year of recordings in Farm A (intensive).

	Farm A (Intensive Farm)							
	New Cases (n)						Cumulative Incidence (%)	
	Year 1			Year 2			Year 1	Year 2
	RS2	RS3	RS4	RS2	RS3	RS4		
Lameness	3	0	1	0	0	0	4.0 (4/100)	0.0 (0/101)
Overgrown hooves	19	7	0	9	4	9	26.8 (26/97)	31.0 (22/71)
Anemia	NA	1	5	6	10	2	6.0 (6/100)	18.9 (18/95)
Mouth scabs, papules	21	1	0	0	0	0	22.0 (22/100)	0.0 (0/103)
Jaw swelling	3	4	3	1	2	1	10.0 (10/100)	3.9 (4/102)
Nasal discharge	0	5	0	1	4	27	5.0 (5/100)	31.1 (32/103)
Udder fibrosis	13	12	15	13	7	18	44.9 (40/89)	55.1 (38/69)
Udder asymmetry	34	25	8	12	11	15	79.8 (67/84)	50.0 (38/76)
Swollen supra-mammary lymph nodes	1	8	0	17	10	2	9.8 (9/92)	42.0 (29/69)
Fecal soiling	1	0	1	0	0	6	2.0 (2/99)	5.9 (6/102)
Poor hair coat quality	3	13	13	13	3	9	41.4 (29/70)	29.4 (25/85)
Body abscesses	30	9	8	8	16	11	51.1 (47/92)	48.6 (35/72)
Swollen body lymph nodes (at least one)	NA	13	9	NA	8	10	25.9 (22/85)	24.7 (18/73)

RS2: April (70 days post-weaning), RS3: June (120 days post-weaning), RS4: August (170 days post-weaning); NA: not applicable (no recording was performed). Calculations were based on a complete dataset from 100 goats in year 1 and 103 goats in year 2. Cumulative incidence values of arthritis, ocular discharge, cough, abnormal respiration, teat cysts, teat fibrosis, signs of acute clinical intramammary infection, udder skin lesions, udder abscesses, udder cysts, vaginitis, and meteorism were below 3.0%.

**Table 6:** New cases and cumulative incidence (total number of new cases/goat population at risk at the beginning) of the studied health and welfare issues per recording session during the first and second year of recordings in Farm B (extensive).

	Farm B (Extensive Farm)							
	New Cases (n)						Cumulative Incidence (%)	
	Year 1			Year 2			Year 1	Year 2
	RS2	RS3	RS4	RS2	RS3	RS4		
Lameness	1	1	0	0	0	0	1.7 (2/115)	0.0 (0/81)
Overgrown hooves	3	2	0	2	2	0	4.3 (5/115)	4.9 (4/81)
Anemia	NA	40	15	18	8	5	50.5 (55/109)	67.4 (31/46)
Jaw swelling	0	1	2	1	0	0	2.6 (3/115)	1.2 (1/81)
Nasal discharge	0	1	16	3	4	20	14.8 (17/115)	47.4 (27/57)
Teat cysts	1	4	1	2	1	0	5.8 (6/104)	3.9 (3/76)
Udder abscesses	3	1	2	0	3	0	5.6 (6/108)	4.1 (3/73)
Udder cysts	4	6	0	0	0	0	8.8 (10/114)	0.0 (0/80)
Udder fibrosis	26	28	22	29	13	13	67.9 (76/112)	76.4 (55/72)
Udder asymmetry	46	17	31	18	12	7	91.3 (94/103)	50.7 (37/73)
Swollen supra-mammary lymph nodes	3	5	6	18	7	9	14.3 (14/98)	54.8 (34/62)
Fecal soiling	4	4	3	0	0	1	9.6 (11/114)	1.2 (1/81)
Poor hair coat quality	9	11	6	5	3	1	44.1 (26/59)	18.4 (9/49)
Body abscesses	17	12	9	6	9	9	43.7 (38/87)	35.8 (24/67)
Swollen body lymph nodes (at least one)	NA	14	11	NA	16	7	32.1 (25/78)	51.1 (23/45)

RS2: April (70 days post-weaning), RS3: June (120 days post-weaning), RS4: August (170 days post-weaning); NA: not applicable (no recording was performed). Calculations were based on a complete dataset from 115 goats in year 1 and 81 goats in year 2. Cumulative incidence values of arthritis, mouth scabs and papules, ocular discharge, cough, abnormal respiration, teat fibrosis, signs of acute clinical intramammary infection, udder skin lesions, vaginitis, and meteorism were below 3.0%.

**Table 7:** Effects of the studied health and welfare issues on daily milk yield.

					95% Confidence Interval	
	EMM	B-Coefficient ^†^	SE	Significance	Lower	Upper
Lameness	1.35	−0.25	0.123	0.040	−0.50	−0.01
Arthritis	1.34	−0.13	0.220	0.562	−0.56	0.30
Overgrown hooves	1.35	−0.09	0.046	0.059	−0.18	0.00
Anemia	1.33	−0.03	0.031	0.347	−0.09	0.03
Mouth scabs, papules	1.35	−0.19	0.081	0.020	−0.35	−0.03
Jaw swelling	1.34	−0.13	0.084	0.115	−0.03	0.30
Ocular discharge	1.34	0.16	0.163	0.340	−0.16	0.48
Cough	1.35	−0.35	0.228	0.127	−0.79	0.10
Nasal discharge	1.34	0.02	0.043	0.690	−0.07	0.10
Teat cysts	1.35	0.05	0.084	0.573	−0.12	0.21
Teat fibrosis	1.35	0.18	0.465	0.696	−0.73	1.09
Signs of acute clinical intramammary infection	1.35	-	-	-	-	-
Udder lesions	1.35	0.05	0.164	0.779	−0.28	0.37
Udder abscesses	1.34	0.09	0.074	0.230	−0.06	0.23
Udder cysts	1.34	0.18	0.111	0.107	−0.04	0.40
Udder fibrosis	1.38	−0.13	0.025	<0.001	−0.18	−0.08
Udder asymmetry	1.38	−0.09	0.024	<0.001	−0.13	−0.04
Swollen supra-mammary lymph nodes	1.35	0.00	0.033	0.959	−0.06	0.07
Fecal soiling	1.35	0.00	0.086	0.999	−0.17	0.17
Poor hair coat quality	1.34	0.03	0.029	0.286	−0.03	0.09
Vaginitis	1.35	−0.03	0.304	0.918	−0.63	0.57
Meteorism	1.35	−0.08	0.132	0.530	−0.34	0.18
Body abscesses	1.35	−0.04	0.028	0.145	−0.10	0.01
Swollen body lymph nodes (at least one)	1.29	−0.04	0.032	0.247	−0.10	0.03

^†^ Reference category: goats without the health issue, EMM: estimated marginal mean of goats without the health issue; SE: standard error. The models included a random intercept with their variance estimated at 0.19; analyses were based on 1827 recordings from 286 goats.

## Data Availability

Data are available upon request.

## Abbreviations

The following abbreviations are used in this manuscript:

AWIN	Animal welfare indicators
BCS	Body condition score
CI	Cumulative incidence
DMY	Daily milk yield
EFSA	European Food Safety Authority
EMM	Estimated marginal means
ICAR	Internation committee for animal recordings
IMI	Intramammary infection
NA	Not applicable
PDO	Protected designation of origin
RS	Recording session
RS1	Recording session 1
RS2	Recording session 2
RS3	Recording session 3
RS4	Recording session 4
SE	Standard error
